# Obstruction of Labial Venous Drainage Following Hyaluronic Acid Injection: Two Case Reports and a Review of the Literature

**DOI:** 10.7759/cureus.106373

**Published:** 2026-04-03

**Authors:** Macarena Olivares, Diego Araya, Francisca Torreblanca, María José Benoit, Juan Francisco Herbosa, Victor Mercado

**Affiliations:** 1 Department of Aesthetic Medicine, Instituto Chileno de Rejuvenecimiento y Optimización de Medicina Estética, Santiago, CHL; 2 Department of Aesthetic Medicine, Advanced Aesthetic Academy, Santiago, CHN; 3 Department of Aesthetic Medicine, Advanced Aesthetic Academy, Santiago, CHL; 4 Department of Aesthetic Medicine, Clínica Herbosa (HEOS), Santiago, CHL; 5 Departmentt of Otolaryngology, Instituto de Neurorrehabilitación y Equilibrio, Viña del Mar, CHL

**Keywords:** hyaluronic acid filler, lip augmentation, perioral anatomy, vascular adverse event, venous congestion, venous outflow obstruction

## Abstract

Vascular adverse events associated with hyaluronic acid (HA) dermal fillers are typically interpreted as being of arterial origin, while venous involvement of the lip remains poorly characterized. The aim of this report is to analyze and update vascular events of venous origin, particularly within the anatomical territory of the lips. We present two cases of young women who developed an acute increase in lip volume with violaceous congestion shortly after perioral HA injection. The clinical presentation was more consistent with obstruction of venous drainage rather than classic arterial ischemia. Both patients received immediate treatment with hyaluronidase on the same day, followed by close clinical monitoring, achieving complete resolution without tissue loss in approximately five days. Recent perioral venous mapping studies have demonstrated variable labial tributaries and well-defined zones of venous accumulation, supporting the presence of an anatomical substrate for this presentation. These cases highlight a distinct post-filler phenotype that should be differentiated from arterial occlusion, angioedema, and hematoma in order to allow timely treatment and prevent progression to irreversible tissue injury.

## Introduction

Hyaluronic acid (HA) fillers are among the most commonly performed minimally invasive aesthetic procedures worldwide due to their reversibility, relative safety, and predictable cosmetic outcomes [1-3]. However, vascular adverse events remain one of the most feared complications of facial filler injections [4-6]. Most published reports and treatment algorithms have focused on arterial occlusion, whether caused by direct intravascular embolization or external compression of an arterial branch, since arterial involvement may lead to ischemia, tissue necrosis, blindness, or stroke [4-9].

In contrast, venous involvement in the perioral region remains insufficiently characterized. Recent studies on perioral venous mapping have demonstrated that venous tributaries are consistently present but anatomically variable, with several well-defined zones of venous accumulation overlapping common lip injection sites [10]. Specifically, venous tributaries have been identified superior to the upper vermilion border, between the nasolabial fold and the philtral column, as well as approximately 1-1.5 cm lateral to the oral commissures, extending inferiorly toward the chin and the labiomental fold. Additional venous accumulation zones have been described approximately 2 mm superior to the Cupid’s bow, along the central tubercle of the upper lip, at the junction between the wet and dry zones of the lower lip, and centrally between the tubercles of the lower lip [10].

Clinically, venous congestion in other vascularized tissues has been described as a dark blue-purple discoloration associated with increased tissue temperature and turgor, whereas arterial insufficiency is characterized by pallor, coolness, and absence of bleeding on pinprick testing [11]. Although this evidence originates from reconstructive microsurgery rather than filler-related complications, it provides a useful clinical framework for interpreting facial vascular compromise when edema and congestive discoloration predominate without the classic signs of arterial ischemia.

We present two cases of acute vascular compromise of the lip following HA filler injection in which the clinical phenotype was compatible with obstruction of venous drainage rather than classical arterial occlusion. The aim of this report is to describe their presentation, analyze the differential diagnosis, and explore potential anatomical and pathophysiological mechanisms.

## Case presentation

A retrospective analysis was conducted of two patients who developed acute vascular compromise of the lips following HA dermal filler injection. Clinical presentation, treatment, and outcomes were documented using standardized clinical photography and follow-up evaluation.

Case 1

A 21-year-old woman underwent HA injection for lip augmentation. Shortly after the procedure, she developed a rapid increase in the volume of the entire upper lip, associated with progressive violaceous discoloration.

**Figure 1:**
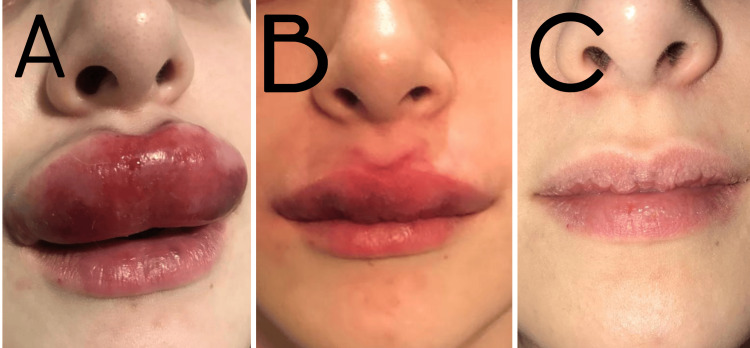
Clinical presentation and resolution of venous-pattern vascular compromise of the upper lip A) Acute venous-pattern vascular compromise characterized by marked edema and diffuse violaceous congestion of the upper lip; (B) Immediate clinical improvement was observed following the administration of 1,500 IU of hyaluronidase; (C) Complete resolution eight days after treatment, without evidence of tissue necrosis.

Clinical examination revealed marked edema and diffuse congestive discoloration involving the entire upper lip. The tissue appeared dark and tense, without pallor, livedoid reticulation, or other features typically associated with arterial ischemia. Capillary refill was preserved. The patient reported moderate discomfort but not the severe ischemic pain commonly observed in acute arterial occlusion. Prior to initiation of the high-dose pulsed hyaluronidase protocol, the patient received antihistamines and corticosteroids without clinical improvement. Based on the distribution of findings, impairment of the upper labial venous drainage was considered a plausible explanation (Figure 1A).

The patient received immediate treatment with a high-dose pulsed protocol with 1,500 IU of hyaluronidase infiltrated throughout the affected area with a 30 G needle; adjunctive anti-inflammatory therapy (Prednisone 20 mg every 12 hours for five days) was initiated; and close clinical monitoring was maintained. Progressive improvement was observed over the following hours (Figure 1B), with complete resolution of swelling and discoloration. No tissue loss or delayed necrosis occurred (Figure 1C).

Case 2

**Figure 2:**
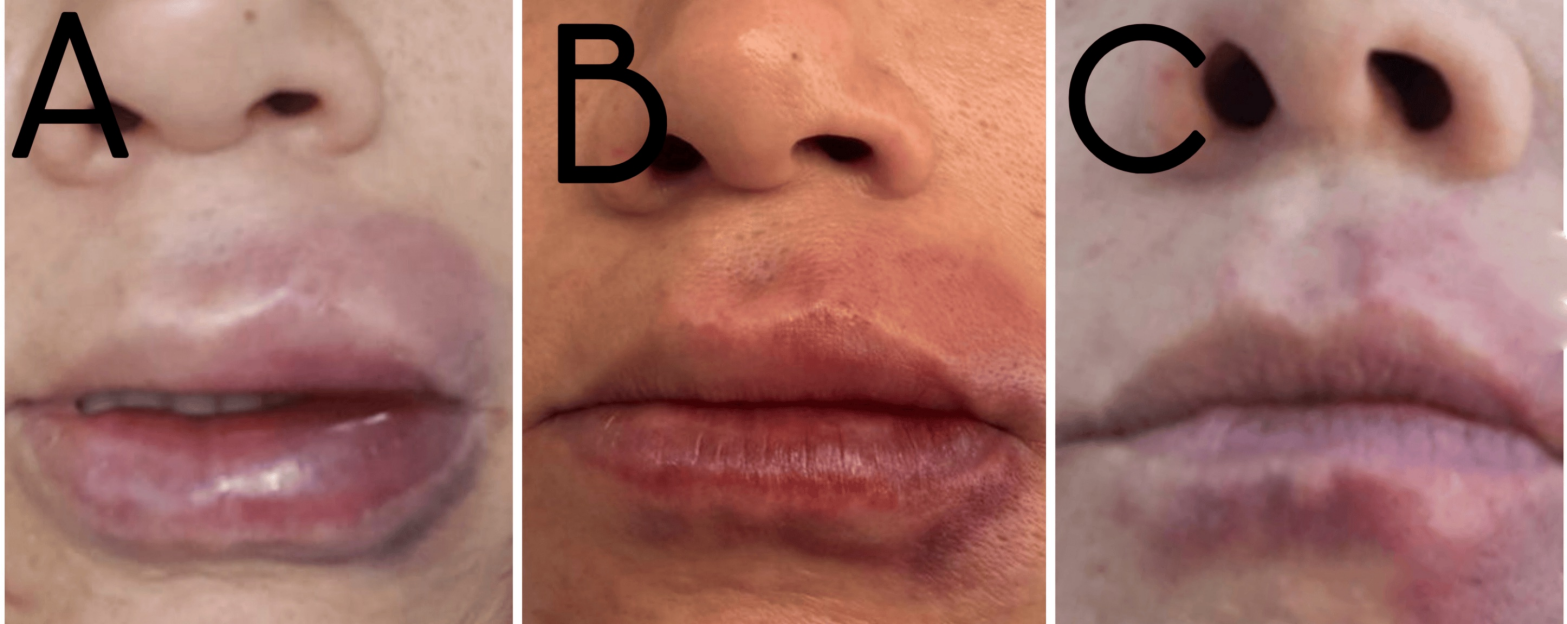
Unilateral venous-pattern vascular compromise affecting the perioral region (A) Localized venous-pattern vascular compromise involving the left upper and lower lips, with edema and violaceous discoloration; (B) Rapid clinical improvement following administration of 1,500 IU of hyaluronidase; (C) Progressive resolution three days after treatment.

A 24-year-old woman underwent HA filler injection in the perioral region. Shortly after treatment, she developed localized swelling and dark violaceous discoloration involving both the upper and lower lips on the left side (Figure 2A).

Clinical examination revealed edema and congestive violaceous discoloration limited to the left perioral tissues. Capillary refill remained preserved, and no pallor or livedoid pattern was observed. As in the first case, the clinical presentation did not match the typical phenotype of arterial ischemia. The simultaneous involvement of the ipsilateral upper and lower lips suggested the possibility of an anatomical venous variant with shared or convergent venous drainage.

Treatment with 1,500 IU of hyaluronidase was administered on the same day, distributed throughout the affected region, while considering the possible anatomical variation. Rapid clinical improvement was observed (Figure 2B), with progressive resolution of edema (Figure 2C) in the following days.

## Discussion

Vascular complications following HA filler injection are traditionally interpreted within an arterial framework, as arterial occlusion represents the most documented and severe vascular adverse event associated with dermal fillers [4-9]. However, the cases presented in this report did not follow the classical pattern suggestive of acute arterial vascular compromise of the lips.

In both patients, the predominant clinical findings included rapid edema, dark violaceous discoloration, preserved capillary refill, and absence of pallor or livedoid reticulation. This phenotype is more consistent with impaired venous drainage rather than classical arterial insufficiency.

**Figure 3:**
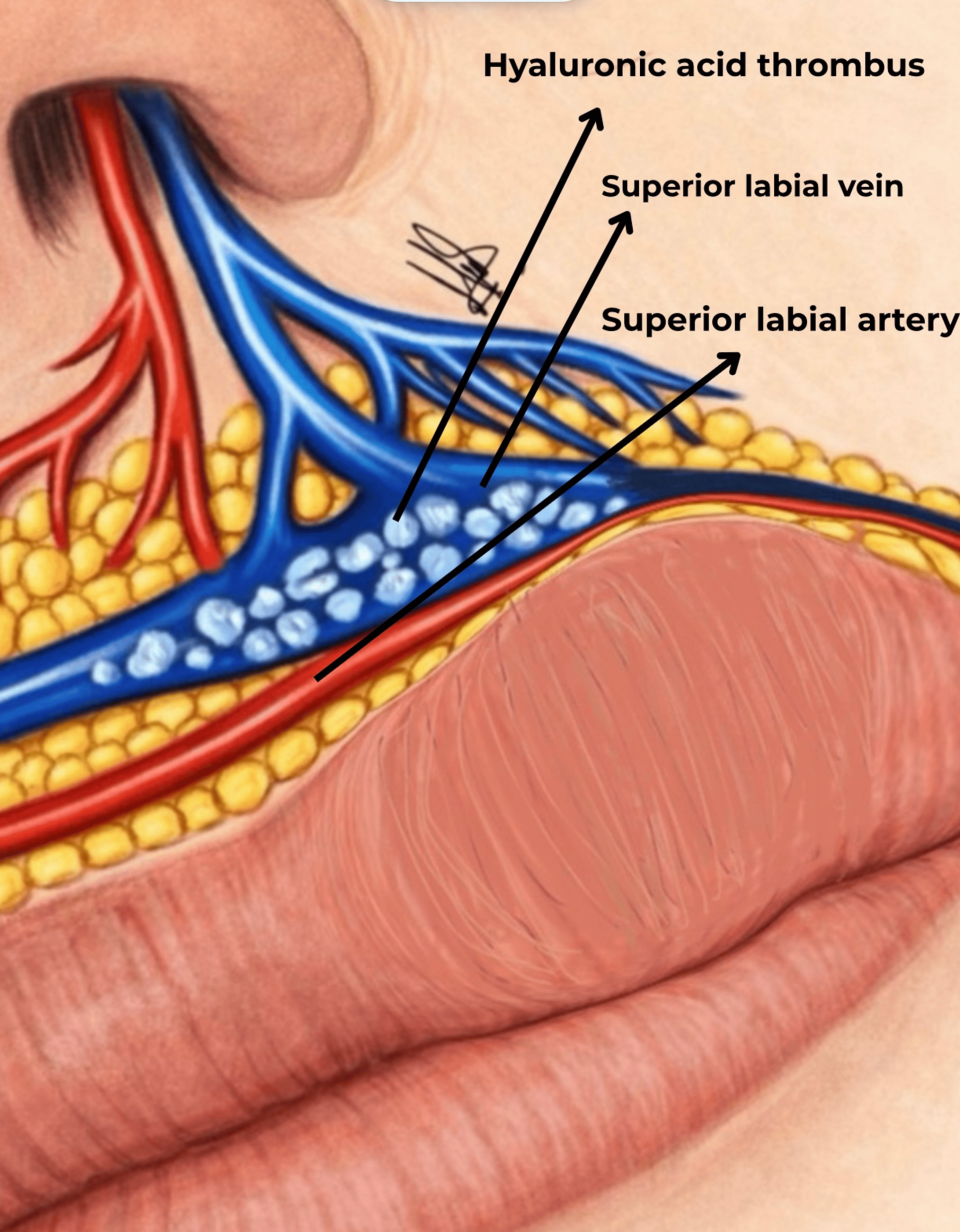
Schematic illustration of the superior labial vascular anatomy Anatomical vascular lip illustration showing the superior labial artery (red) and superior labial vein (blue). A hyaluronic acid thrombus is depicted within the venous lumen, illustrating intravascular filler accumulation and its potential role in vascular compromise and impaired venous outflow in the upper lip region. Illustration by Macarena Olivares. This figure was hand-drawn and digitized using Flow Sketchbook (Moleskine S.p.A., Milan, Italy).

The anatomical plausibility of venous involvement is supported by recent perioral venous mapping studies, which demonstrate significant variability in the pattern and distribution of labial venous tributaries (Figure 3) [10]. Moorefield et al. [11] identified several anatomically vulnerable zones of venous accumulation in the lips and surrounding regions, many of which overlap with common filler injection sites. In the first reported case, the involvement of the entire upper lip may reflect impairment of upper labial venous drainage. In the second case, the simultaneous congestion of the ipsilateral upper and lower lips could suggest a venous anatomical variant in which tributaries from both lips converge into a shared drainage pathway.

The clinical phenotype observed also parallels the classical description of venous congestion reported in reconstructive microsurgery. In flap surgery, venous compromise typically manifests as dark purple or bluish discoloration associated with increased tissue temperature and turgor, whereas arterial compromise presents with pallor, coldness, and diminished capillary refill (Table 1) [11,12]. This comparison provides a clinically coherent framework for interpreting venous-pattern congestion following filler injection.

**Table 1:** Comparative clinical features of arterial occlusion versus venous outflow obstruction following hyaluronic acid filler injection Arterial occlusion is characterized by ischemic features, including pallor, reduced capillary refill, and high risk of tissue necrosis. In contrast, venous outflow obstruction presents with violaceous congestion, preserved capillary refill, and rapid edema formation. A prompt reduction in edema and volume following hyaluronidase administration is suggestive of a venous-pattern vascular event.

Feature	Arterial Occlusion	Venous Obstruction
Primary mechanism	Intravascular embolization or external arterial compression	Impaired venous outflow (compression or intravascular obstruction)
Onset	Immediate (seconds–minutes)	Immediate to early (minutes–hours)
Color	Pallor → livedo reticularis → dusky gray	Violaceous/bluish congestion
Temperature	Cold	Normal to warm
Pain	Severe, sharp, disproportionate	Mild to moderate, pressure-like or tension
Capillary refill	Delayed or absent	Normal or slightly delayed
Bleeding on a pinprick	Absent	Present (dark, deoxygenated blood)
Edema/volume	Minimal initially, increases later with necrosis	Marked, rapid edema and volumetric increase
Skin turgor	Decreased (ischemic)	Increased (congestive)
Progression	Rapid progression to ischemia and necrosis	Usually self-limited if treated; low risk of necrosis if early management
Tissue oxygenation	Severely compromised	Relatively preserved initially
Typical distribution	Follows arterial territories	Diffuse or dependent congestion patterns
Response to hyaluronidase	Variable; requires high doses and repeated injections	Rapid improvement with immediate reduction in edema/volume
Time to clinical improvement	Hours to days	Often minutes to hours
Risk of necrosis	High, if untreated	Low, if promptly treated
Associated complications	Blindness, stroke (rare but severe)	Rare progression to necrosis, if misdiagnosed

**Figure 4:**
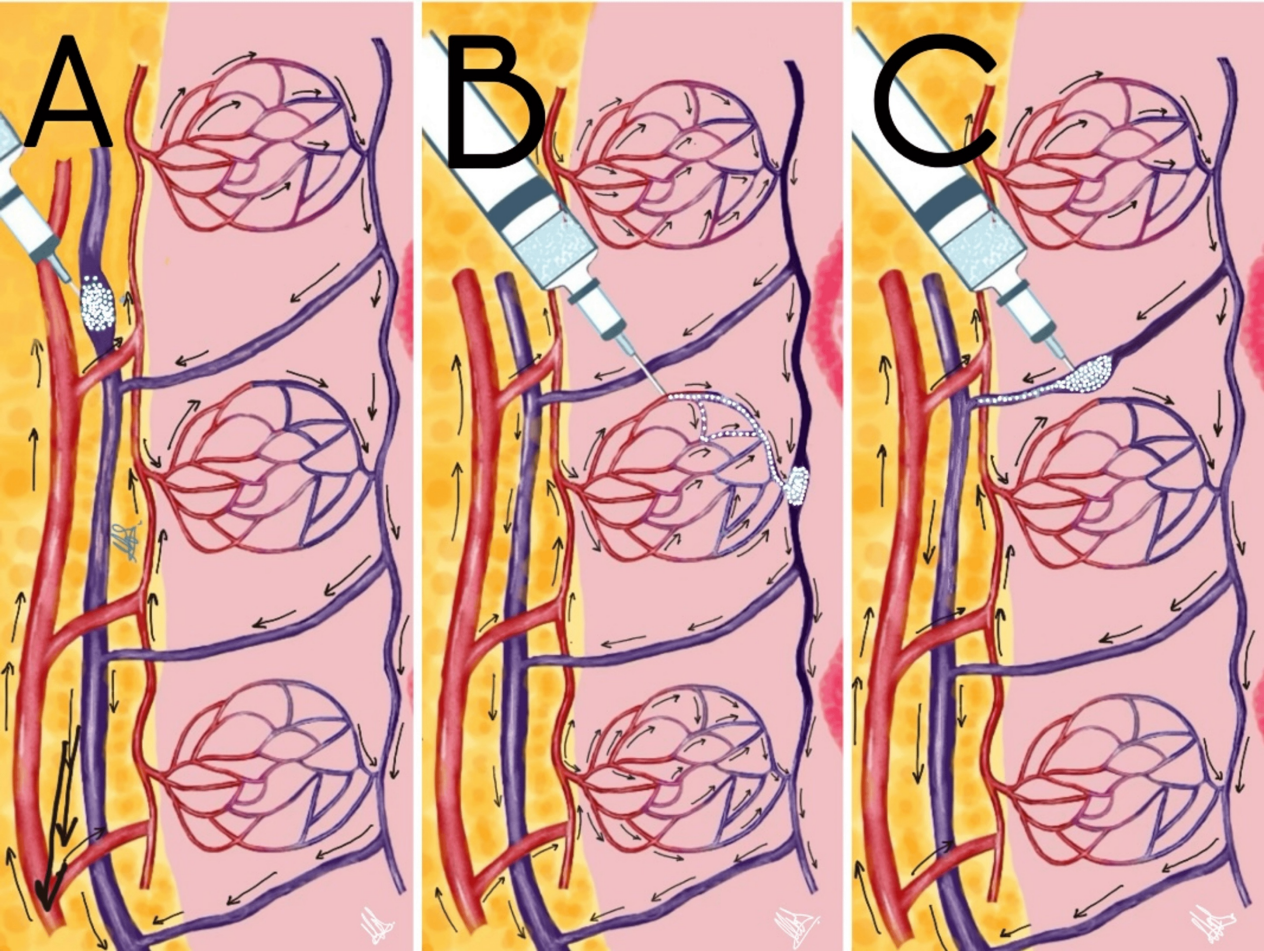
Proposed mechanisms underlying venous outflow obstruction following hyaluronic acid injection (A) Direct intraluminal injection into a labial venous tributary; (B) Involvement of the microvascular/capillary plexus with secondary venous pooling; (C) Injection into the perioral venous plexus during filler administration. Illustration by Macarena Olivares. This figure was hand-drawn and digitized using Flow Sketchbook (Moleskine S.p.A., Milan, Italy).

Several mechanisms could explain venous vascular compromise following HA injection. One possibility is direct intraluminal injection into a labial venous tributary during filler placement. Another potential mechanism involves a mixed microvascular event in which both arterial and venous components are present, although the venous component predominates clinically. A third possibility is inadvertent injection into the perioral venous plexus during filler administration. These mechanisms are not mutually exclusive and are illustrated in Figure 4 [11-16].

Experimental studies have demonstrated that HA gels may fragment and disperse following intravascular injection, although these models primarily involve arterial systems and do not directly demonstrate venous embolization [17,18]. Their relevance lies in supporting the broader concept that HA may behave dynamically once introduced into the vascular lumen. Therefore, the term "venous flow obstruction pattern" may be more appropriate than "definitive venous embolism" in the absence of imaging or histopathological confirmation.

An important clinical implication of these cases is the need for careful differential diagnosis. A vascular event with a venous pattern may be mistaken for post-procedural edema, angioedema, or hematoma. Conversely, assuming that all vascular changes following filler injection are arterial may oversimplify events whose clinical presentation is dominated by venous stasis. Preserved capillary refill, absence of pallor, lack of livedoid reticulation, and predominance of violaceous congestion should raise suspicion for venous compromise [15-16].

The favorable outcome observed following immediate high-dose pulsed hyaluronidase protocol administration should not be interpreted as confirmation of the underlying mechanism but rather supports the importance of early treatment in any suspected HA-related vascular event.

This study has limitations. Only two cases were analyzed, and Doppler ultrasound, angiography, or histopathological confirmation of venous obstruction was not available. Therefore, the proposed diagnosis remains clinical-anatomical and inferential. Further research integrating imaging studies, anatomical correlation, and prospective case collection is required to clarify whether post-filler venous compromise represents true venous embolism, external venous compression, or a mixed vascular pleno menonitas.

## Conclusions

Venous outflow obstruction of the lips may represent an underrecognized pattern of vascular compromise associated with HA filler injections. Unlike classical arterial occlusion, this presentation is characterized by rapid swelling, violaceous congestion, preserved capillary refill, and absence of pallor or livedoid reticulation. The anatomical variability of labial venous tributaries and the presence of defined zones of perioral venous pooling provide a plausible anatomical basis for this phenotype. Early recognition and prompt treatment remain essential to prevent progression to tissue injury. Further anatomical, imaging, and clinical studies are needed to better define the mechanisms and clinical significance of venous-pattern vascular events following lip filler injections.
